# Antitumorigenic Effect of Tramadol and Synergistic Effect With Doxorubicin in Human Breast Cancer Cells

**DOI:** 10.3389/fonc.2022.811716

**Published:** 2022-01-26

**Authors:** Yi-Hsuan Huang, Sung-How Sue, Zih-Syuan Wu, Shih-Ming Huang, Shih-Yu Lee, Zhi-Fu Wu

**Affiliations:** ^1^ Department of Anesthesiology, Tri-Service General Hospital, National Defense Medical Center, Taipei, Taiwan; ^2^ Department of Cardiovascular Surgery, Hsinchu Mackay Memorial Hospital, Hsinchu, Taiwan; ^3^ Department of Biochemistry, National Defense Medical Center, Taipei, Taiwan; ^4^ Graduate Institute of Aerospace and Undersea Medicine, National Defense Medical Center, Taipei, Taiwan; ^5^ Department of Anesthesiology, Kaohsiung Medical University Hospital, Kaohsiung Medical University, Kaohsiung, Taiwan; ^6^ Department of Anesthesiology, Faculty of Medicine, College of Medicine, Kaohsiung Medical University, Kaohsiung, Taiwan; ^7^ Center for Regional Anesthesia and Pain Medicine, Wan Fang Hospital, Taipei Medical University, Taipei, Taiwan

**Keywords:** breast cancer, doxorubicin, epithelial–mesenchymal transition, HIF-1α, tramadol

## Abstract

**Background:**

Breast cancer in women is one of the leading causes of cancer mortality worldwide, and curative therapy is the main focus of clinical treatment. Anesthetic-analgesic techniques might alter stress responses and immunity and thereby influence outcomes in cancer patients. This study investigated the effect of tramadol on breast cancer progression and metastasis.

**Methods:**

The effects of tramadol on two different subtypes of human breast adenocarcinoma cell lines, MDA-MB-231 and MCF-7, were studied with regard to cell growth, migration, colony formation and invasion and normoxic or hypoxic microenvironment for the expression of hypoxia-inducible factor-1α, reactive oxygen species, epithelial-mesenchymal transition related and cyclin-related proteins. The co-administration of tramadol and doxorubicin was studied to determine whether the effective doxorubicin dose might be reduced in combination with tramadol.

**Results:**

The results showed that tramadol inhibited cell growth at concentrations more than 0.5 and more than 1.0 mg/mL in MDA-MB-231 and MCF-7 cells, respectively. Additionally, cell migration, colony formation and invasion were inhibited in a dose-dependent manner by tramadol in both cell lines. The combination of tramadol and doxorubicin induced synergistic effects in MDA-MD-231 cells and, with specific dosage combinations in MCF-7 cells.

**Conclusions:**

Tramadol may regulate epithelial-mesenchymal transition and possess cytotoxic effects in breast cancer cells. Tramadol inhibits the progression of breast cancer cells and might be a candidate for combination therapy, especially for triple-negative breast cancer, and is a promising treatment strategy for breast cancer.

## Introduction

Breast cancer in women, which contributed to 11.7% of the global cancer incidence and 6.9% of global cancer mortality in 2020 (GLOBOCAN report), has surpassed lung cancer as the commonest malignancy and is one of the top five causes of cancer mortality. (1) Surgical resection is one of the major treatment options for breast cancer, and perioperative surgical and anesthetic interventions may alter the stress responses and immunity and could even modulate the tumor microenvironment of patients. (2) The mechanisms through which anesthetic-analgesic techniques influence breast cancer outcomes have increasingly garnered attention although the results of research have been inconsistent. (3–11)

Tramadol is a centrally acting analgesic that is widely accepted in the treatment of moderate postoperative pain. (12) Piñero and colleagues (13) reported that β-adrenoceptor agonists and α_2_-adrenoceptor antagonists can effectively suppress breast cancer cell proliferation and tumor growth *via* the inhibition of extracellular signal-regulated kinase 1/2 (ERK 1/2) phosphorylation in an animal model. Tramadol inactivates α_2_-adrenoceptor signaling and inhibits the proliferation, migration and invasion of breast cancer cells. (14) Kim and co-workers (8) reported that postoperative tramadol use mitigated the risk of cancer recurrence and improved survival in patients with breast cancer. Also, *in vitro* attenuation of the 5-hydroxytryptamine (HT)_2B_ receptor activity and transient receptor potential vanilloid-1 (TRPV1) inhibited tumor growth and promoted apoptosis.

With regard to cancer survival and metastasis, epithelial-mesenchymal transition (EMT) plays a crucial role in the dissemination of cancer cells. (15) EMT is a cellular process wherein epithelial cancer cells are converted to motile mesenchymal cancer cells that trigger metastatic capability. Furthermore, hypoxia provokes EMT, which increases motility, tumorigenesis and, eventually, distant metastasis. (16) The hypoxic microenvironment plays an important role in breast cancer progression and metastasis. (16, 17) However, the anti-tumorigenic effect of tramadol and EMT on breast cancer has not been elucidated.

This study was conducted with an aim to ascertain the effects of tramadol on cell growth, migration and invasion as well as on EMT in relation to breast cancer recurrence and metastasis. The primary objective was to identify the relationship between tramadol and breast cancer through the evaluation of EMT-associated biomarkers [hypoxia-inducible factor 1 alpha (HIF-1α)] and to examine whether tramadol treatment, in a normoxic or hypoxic microenvironment, affects the expression of HIF-1α, stress-induced reactive oxygen species and EMT- and cyclin-related proteins in the human breast adenocarcinoma cell lines MDA-MB-231 and MCF-7. The secondary objective of this study was to evaluate the feasibility of repurposing combination therapy with tramadol and doxorubicin for breast cancer.

## Materials and Methods

### Cell Culture and Reagents

We used two molecular subtypes of human breast adenocarcinoma [MDA-MB-231 derived from triple-negative breast cancer (TNBC) cells] and MCF-7 (luminal breast cancer cells) to evaluate the effects of tramadol treatment. The MDA-MB-231 (ATCC^®^HTB-26™) and MCF-7 (BCRC-60436) human breast adenocarcinoma cell lines were purchased from the American Type Culture Collection (Manassas, VA, USA) and Bioresource Collection and Research Center (Hsinchu, Taiwan), respectively. All cells were cultured in minimum essential medium (MEM) with 2 mM l-glutamine and Earle’s Balanced Salts that contained 1.5 g/L sodium bicarbonate, 0.1 mM non-essential amino acids, 1.0 mM sodium pyruvate, 10% fetal bovine serum (FBS) and 1% penicillin–streptomycin (Thermo Fisher Scientific, Waltham, MA, USA). Doxorubicin, propidium iodide (PI), thiazolyl blue tetrazolium bromide (MTT), and tramadol were procured from Sigma Aldrich (St. Louis, MO, USA).

### Analysis of Cell Metabolic Activity

The MDA-MB-341 and MCF-7 cells (5×10^3^/well) were seeded in 96-well plates, overnight under 5% CO_2_ at 37°C, and subsequently exposed to different dosages of tramadol or doxorubicin for 24 h. Thereafter, 10 µL MTT solution [dissolved in phosphate-buffered saline (PBS) to obtain a concentration of 5 mg/mL] per well was added, and the cells were incubated for at 37°C for at least 1 h. After gently removing the MTT medium, plates were washed twice with PBS and 100 μL dimethyl sulfoxide (DMSO) was added to dissolve MTT crystals, and the absorbances at 570 and 650 nm were measured using an enzyme-linked immunosorbent assay plate reader (Multiskan EX, Thermo Fisher Scientific). CalcuSyn (Biosoft, Cambridge, UK) was used to calculate the combination index (CI) to generate an isobologram (CI <1 and >1 indicates a synergistic and an antagonistic combination effect, respectively). (18)

### Cell-Cycle Profiles

Cells were fixed in 70% ice-cold ethanol and stored at −20°C overnight, then centrifuged (1,000 rpm for 5 min), washed twice with ice-cold PBS supplemented with 1% FBS and stained with PI solution (5 μg/mL PI in PBS, 0.5% Triton X-100, and 0.5 μg/mL RNase A) for 30 min at 37°C in the dark. For each test condition, we collected 10,000 cells for flow cytometry (BD FACSCalibur™) and Cell Quest Pro software (BD Biosciences, Franklin Lakes, NJ, USA).

### Wound-Healing Assay

Cells (3 × 10^5^ cells/well) were seeded in a 24-well plate and incubated for 24 h under 5% CO_2_ at 37°C to form a confluent monolayer. Next, a sterile 200-μL pipette tip was used to vertically draw a cross in each well, and the cells in each well were treated with different tramadol concentrations. After wounding (0 h) and at 16 h post-wounding, the scratch closure was monitored and imaged using a LeadView 2800AC-FL microscope (Leader Scientific Co. Ltd., Taiwan) that was equipped with a 40× objective; the change in the wound area was measured using ImageJ (NIH, Bethesda, MD).

### Colony-Formation Assay

Cells (2 × 10^3^/well) were seeded into six-well plates for 24 h, incubated with different tramadol concentrations for 2 weeks and the colonies that formed were fixed with methanol and stained with 0.005% crystal violet. Colonies that were larger than 0.05 mm were numbered using ImageJ software (NIH, Bethesda, MD).

### Invasion Assay

The invasion assay was performed in Transwell chambers coated with Matrigel matrix (BD Biosciences, San Jose, CA). The cells were added into serum-free MEM in the upper chambers, and MEM containing 10% FBS was added to the lower chambers. The cells were incubated in a 5% CO_2_ incubator at 37° C for 16 h, followed by the removal of the non-migrated cells from the upper chamber. Each chamber was stained with 0.1% crystal violet after fixing with 3.8% formaldehyde in PBS, and the cells were counted under a microscope (10x objective).

### Hypoxic Treatment

MDA-MB-341 and MCF-7 cells (5×10^5^/well) were seeded in six-well plates and cultured at 37°C in 5% CO_2_ for 24 h. On the second day, the culture medium was replaced with fresh medium and the cells were placed in a hypoxia chamber (in a gas mixture comprising 1% O_2_ and 90% N_2_/5% CO_2_) or in a normal incubator after treatment with various tramadol concentrations for 4 h.

### Western Blotting

Cells were washed twice with ice-cold PBS and lysed in a radioimmunoprecipitation assay buffer [100 mM Tris-HCl (pH 8.0), 150 mM NaCl, 0.1% SDS, and 1% Triton X-100] at 4°C. The proteins in the resulting lysate were separated using sodium dodecyl sulphate polyacrylamide gel electrophoresis, and the resolved proteins were immunoblotted with antibodies against β-actin, nuclear factor erythroid 2-related factor 2 (Nrf2), B-cell lymphoma 2 (BCL2) and adenovirus E1B 19-kDa-interacting protein 3 (BNIP-3), p53, Slug (Santa Cruz Biotechnology, Santa Cruz, CA, USA), HIF-1α, α-Smooth muscle actin (α-SMA), transforming growth factor-β (TGF-β), N-cadherin, E-cadherin, Snail, vimentin, poly (ADP-ribose) polymerase (PARP; Cell Signaling Technology, Danvers, MA, USA), γH2A.X, cyclin D1, collagen-I (Abcam, Cambridge, UK), heme-oxidase 1 (HO-1; Enzo Life Sciences, Farmingdale, NY, USA), and differentiated embryonic chondrocyte gene 1 (DEC-1; Bethyl Laboratory, TX, USA).

### Statistical Analysis

Values are expressed as the mean ± SD from at least three independent experiments. The Student’s *t*-tests was used for all intergroup comparisons. Statistical significance was set at *p <*0.05.

## Results

### Tramadol Impeded MDA-MB-231 and MCF-7 Cell Growth

**Figure 1:**
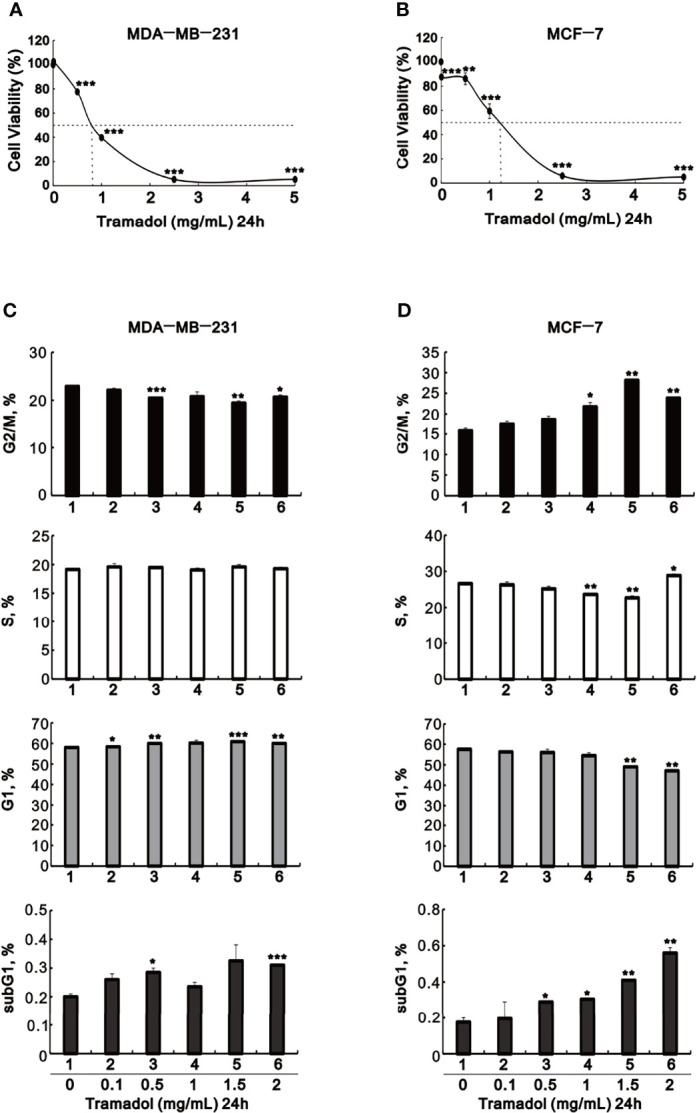
The effects of tramadol on cell viability and the cell-cycle profiles of human breast cancer cells. **(A–D)** MDA-MB-231 and MCF-7 cells were treated with tramadol (0, 0.1, 0.5, 1.0, 2.5 and 5 mg/mL) for 24 h. **(A, B)** Cell viability was measured according to the MTT method. **(C, D)** For cell-cycle profiles, the cells were stained with propidium iodide (PI) and analyzed by flow cytometry. Bars depict the mean ± SD of three independent experiments. *p < 0.05, **p < 0.01 and ***p < 0.001 (Student’s *t*-tests).

To verify the impact of tramadol on MDA-MB-231 and MCF-7 cells, cell viability assay experimented in cultures treated by tramadol at concentration ranging from 0.01 to 5 mg/mL for 24 hr. The growth of MDA-MB-231 and MCF-7 cells were significantly suppressed at tramadol concentration more than 0.5 and more than 1.0 mg/mL (**Figures 1A, B**), respectively, indicating a dose-dependent inhibition of cell growth following treatment with tramadol in both cell lines. The results showed that the tramadol half-maximal inhibition concentrations (IC_50_) were determined as 0.8 and 1.1 mg/mL for MDA-MB-231 and MCF-7, respectively. After treatment with the indicated tramadol concentrations for 24 h, the distribution of MDA-MB-231 and MCF-7 cells in different cell-cycle phases were examined. The MDA-MB-231 cells demonstrated a significant dose-dependent decreased in the G2/M phase population and a dose-dependent increase in the G1 phase population (**Figure 1C**). In contrast, there was a significant dose-dependent increase in the G2/M and a significant dose-dependent decreased in the G1 phase populations of MCF-7 cells following treatment with tramadol (**Figure 1D**). Moreover, the sub-G1 phase population showed a significant but slight increase in MCF-7 cells.

### Tramadol Suppressed Migration, Colony Formation and Invasion of MDA-MB-231 and MCF-7 Cells

**Figure 2:**
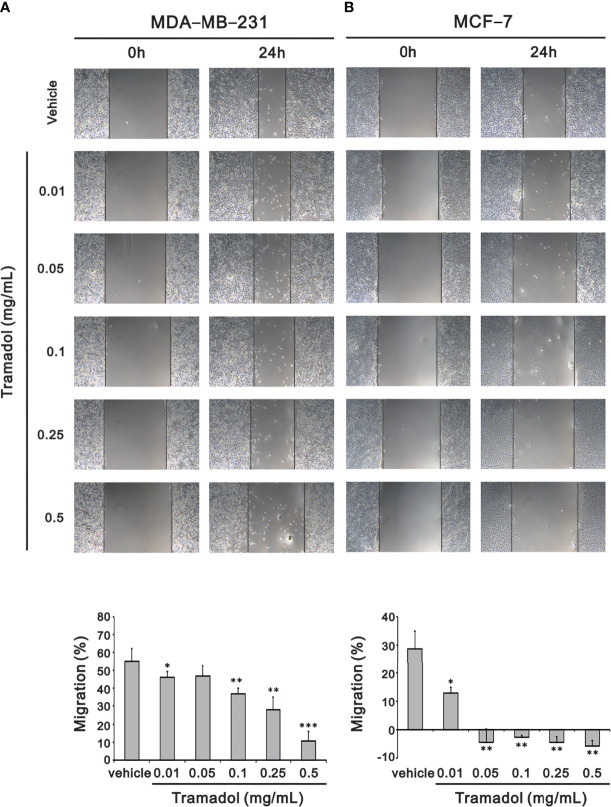
Analysis of the effect of tramadol on the cell migration of human breast cancer cells in a wound-healing assay. **(A, B)** MDA-MB-231 and MCF-7 cells were treated with tramadol (0, 0.01, 0.05, 0.1, 0.25 and 0.5 mg/mL) for 24 h. Quantification of the migration area of ​​untreated and tramadol-treated cells within 24 h using Image J Bars depict the mean ± SD of three independent experiments. *p < 0.05, **p < 0.01 and ***p < 0.001 (Student’s *t*-tests).

Recurrence and metastases of breast cancer are the key elements of cancer outcomes and related to cancer survival, among which migration of cancer cells plays an important role in metastases. To determine the impact of tramadol on breast cancer cell migration, a wound-healing assay was measured to assess the migration rates. After 24-h treatment with tramadol (concentration more than 0.01 mg/mL), the rate of migration decelerated significantly in MDA-MB-231 cells (**Figure 2A**); in contrast, the suppressive effect of 24-h tramadol treatment (concentration more than 0.1 mg/mL) on the rate of migration was significantly slower in MCF-7 cells (**Figure 2B**).

**Figure 3:**
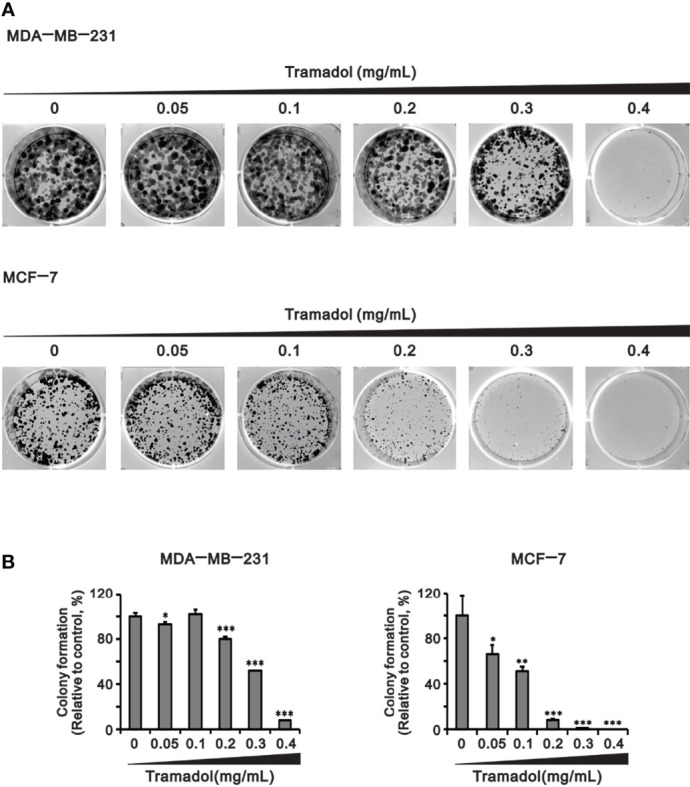
Analysis of the effect of tramadol on the colony-formation ability of human breast cancer cells. **(A, B)** MDA-MB-231 and MCF-7 cells were treated with tramadol (0, 0.05, 0.1, 0.2, 0.3 and 0.4 mg/mL) for 14 days. Bars depict the mean ± SD of three independent experiments. * *p <*0.05, ** *p <*0.01 and *** *p <*0.001 (Student’s *t*-tests). Bars depict the mean ± SD of three independent experiments.

Colony formation was examined by evaluation of the size of the colony to identify the impact of anchorage-independent growth by tramadol. Compared to the control groups, colony formation of MDA-MB-231 and MCF-7 cells was significantly inhibited following treatment with tramadol (concentration more than 0.2 and more than 0.05 mg/mL; **Figures 3A, B**), respectively. Tramadol inhibited colony formation in both cell lines in a dose-dependent manner (**Figure 3**).

**Figure 4:**
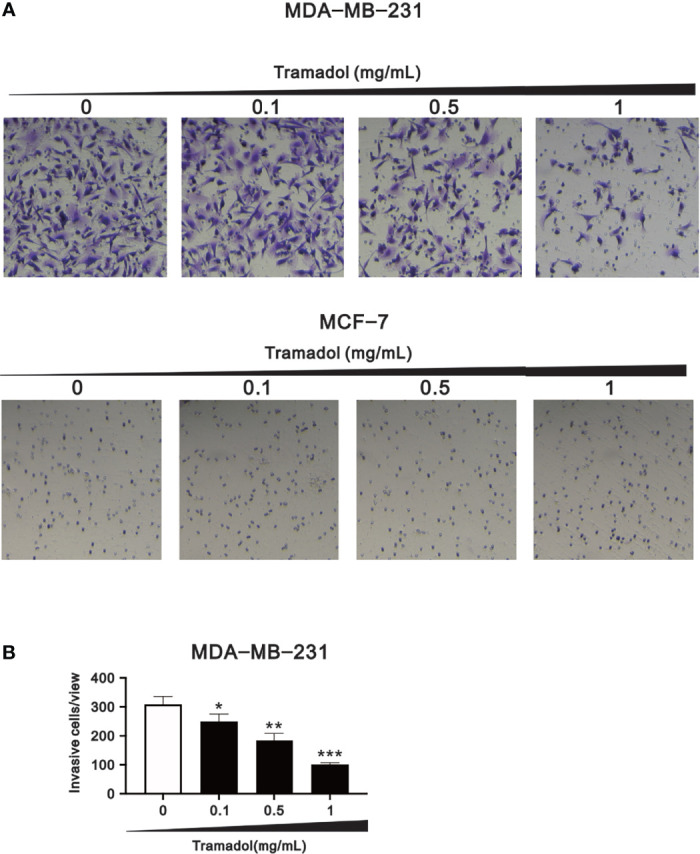
Analysis of the effect of tramadol on the invasiveness of human breast cancer cells. **(A, B)** MDA-MB-231 and MCF-7 cells were treated with tramadol (0, 0.1, 0.5 and 1 mg/mL) for 24 h. Bars depict the mean ± SD of three independent experiments. *p < 0.05, **p < 0.01 and ***p < 0.001 (Student’s *t*-tests). Bars depict the mean ± SD of three independent experiments.

To determine the impact of tramadol on breast cancer cell invasiveness, the amounts of invasive cells were examined using the trans-well assay. The invasiveness of MDA-MB-231 cells was significantly attenuated following tramadol treatment (concentration more than 0.1 mg/mL; **Figures 4A, B**), whereas MCF-7 cells showed no invasive capability (**Figure 4A**).

### Effects of Tramadol on Hypoxia, Oxidative Stress, DNA Damage, Cell Death, Cell Cycle and EMT-Related Proteins in MDA-MB-231 and MCF-7 Cells

**Figure 5:**
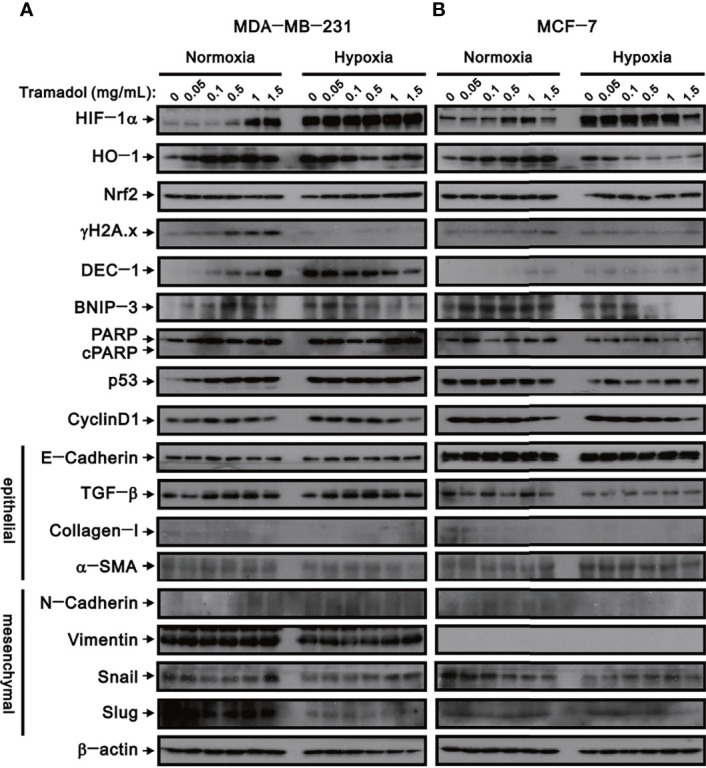
The effects of tramadol on protein expression in human breast cancer cells. **(A, B)** MDA-MB-231 and MCF-7 cells were treated with tramadol (0, 0.05, 0.1, 0.5, 1 and 1.5 mg/mL) for 4 h. β-actin (lower panel) served as the loading control.

Hypoxia facilitates EMT at the very beginning of breast cancer invasion and eventually accomplishes distant metastasis with a poor prognosis. To determine the relationship between tramadol and EMT in breast cancer cell invasion, a series of western blot analyses for protein associated with hypoxia, oxidative stress, DNA damage, cell death, cell cycle, and EMT were measured. Under hypoxic conditions, HIF-1α was highly expressed in MDA-MB-231 cells (**Figure 5A**); following treatment with increasing concentrations of tramadol in normoxic conditions, HIF-1α expression was induced, but no further upregulation of HIF-1α expression was observed under hypoxic conditions. Nrf-2 is a well-known transcription factor that plays a role in the maintenance of the cellular redox balance, and HO-1 is one of the targets of Nrf-2 in the mediation of the intracellular antioxidant function. Similar to the HIF-1α expression following tramadol treatment, HO-1 expression, but not Nrf-2 expression, increased in a dose-dependent manner in a normoxic environment in MDA-DB-231 cells (**Figure 5A**). After the tramadol treatment, the expression of γ-H2A.x, a sensitive marker of DNA double-strand breaks and a potential breast cancer biomarker, (19) increased in the normoxic environment (in a dose-dependent manner) but was undetectable in the hypoxic environment.

The expression of three cell death-related proteins—DEC-1, BNIP3, and cleaved PARP—were examined following tramadol treatment and induction of hypoxia in MDA-MB-231 cells. After tramadol treatment, dose-dependent increases in DEC-1 and BNIP3 expressions in a normoxic environment were observed. However, DEC-1 and BNIP3 expressions that were induced in a hypoxic environment diminished in a dose-dependent manner following tramadol treatment. After tramadol treatment and induction of hypoxia in MDA-MB-231 cells, the expression of cleaved PARP was undetectable, which suggested the absence of apoptosis. To determine the effects of tramadol treatment and hypoxia in MDA-MB-231 cells, we measured the expression of two cell-cycle-related proteins, p53 and cyclin D1. Tramadol and hypoxia separately induced p53 expression but a further enhancement by a combination of the two treatments was absent. Regardless of normoxia or hypoxia, cyclin D1 expression was inhibited by treatment with tramadol. Finally, we examined the expression of proteins related to EMT, including the epithelial markers E-cadherin, TGF-β, α-SMA and collagen I as well as the mesenchymal markers N-cadherin, vimentin, Snail and Slug in MDA-MB-231 cells. Tramadol treatment resulted in the transition of MDA-MB-231 cells into a mesenchymal state *via* the induction of TGF-β and α-SMA and suppression of E-cadherin and collagen I and the induction of N-cadherin, vimentin, Snail and Slug. Under hypoxic conditions, the effect of tramadol on EMT was eliminated.

Simultaneously, the expression of the abovementioned proteins was examined in MCF-7 cells (**Figure 5B**). The effects of tramadol treatment and hypoxia in MCF-7 cells were similar to those in MDA-MB-231 cells except that vimentin proteins were undetectable in MCF-7 cells and p53 expression was not induced by tramadol and was suppressed by hypoxia.

### Synergistic Effect of Tramadol on Doxorubicin-Treated MDA-MB-231 and MCF-7 Cells

**Figure 6:**
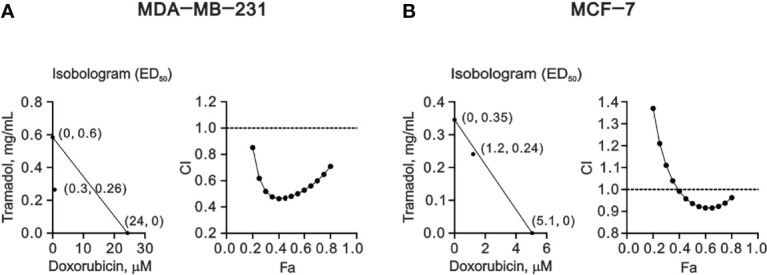
The combination index (CI) of combination treatment of tramadol and doxorubicin in human breast cancer cells. **(A, B)** MDA-MB-231 and MCF-7 cells were treated with tramadol (0, 0.0625, 0.125, 0.25, 0.5, 1, 2 and 4 mg/mL) and doxorubicin (0, 0.0390625, 0.078125, 0.15625, 0.3125, 0.625, 1.25, 2.5, 5 and 10 mM) for 24 h. The experimental points below the line correspond to CI <1, indicating a synergistic effect.

Our current findings suggested that tramadol might be a candidate for the combination therapy for breast cancer, especially for TNBC. Doxorubicin is a common chemotherapy drug applied for TNBC. Hence, we designed various amounts of tramadol and doxorubicin for the combination index analysis **(** **Figure 6**). The CI <1 indicated that all combinations of tramadol and doxorubicin induced synergistic effects in MDA-MD-231 cells (**Figure 6A**). Therefore, combination therapy with tramadol might reduce the effective concentration of doxorubicin from 24 μM to 0.3 μM. Similarly, in MCF-7 cells, combination therapy with tramadol and doxorubicin induced synergistic effects at specific dosage combinations (**Figure 6B**) that facilitated the reduction of the effective doxorubicin concentration from 5.1 to 1.2 μM.

## Discussion

The findings of this study show that tramadol has potential cytotoxic properties and inhibits the migration, colony formation and invasion of breast cancer cells. Furthermore, a synergistic effect of tramadol in combination therapy with doxorubicin in breast cancer cell lines was observed.

Tramadol, which is used for acute pain management after breast cancer surgery, is associated with lower risk of tolerance, dependence and respiratory depression. (20, 21) Recent preclinical and clinical studies have shown that tramadol possesses immunostimulatory effects that through NK cell activation and lymphocyte proliferation (22, 23); moreover, tramadol reduces the risk of lung metastasis in rats. (24) Tramadol confers an anti-tumorigenic effect against proliferation, migration and invasion in lung cancer cells by upregulating the phosphatase and tensin homolog and interfering with phosphoinositide 3-kinase/protein kinase B (PI3K/Akt) signaling (25) and by downregulating the α2-receptor in breast cancer cells (MDA-MB-231) (14); our results are consistent with the abovementioned findings. Tumor resection potentially enhances the emergence and seeding of circulating tumor cells through ischemia-reperfusion injury, activation of the sympathetic nervous system, inflammation, induction of a systemic hypercoagulable state, immunosuppression and the effect of anesthetics. (26) Tramadol has positive effects on antioxidant levels in renal injury and in myocardial ischemia-reperfusion injury. (27, 28) In contrast to morphine, tramadol improved postoperative immunosuppression, which might be a desirable feature in a postoperative pain-management option. (22) Likewise, tramadol suppresses sympathetic nervous activity through the inhibition of nicotinic acetylcholine receptors. (29) Furthermore, tramadol induces hypocoagulable changes in patients with gynecologic cancer and may be useful for patients with an impending hypercoagulable state. (30) In our opinion, tramadol, due to its abovementioned properties and anticancer benefits, confers a superior prognosis for patients with breast cancer in addition to pain relief.

Molecular classifications of breast cancer are characterized as five different subtypes: luminal-A, luminal-B, human epidermal growth factor receptor 2 (HER2)-positive, basal-like and normal breast-like. (31, 32) The basal-like subgroup does not express the estrogen receptor (ER), progesterone receptor (PR) or HER2 and is referred to as triple-negative subtype, which is notorious for its aggressive pattern, a tendency for early relapse and recurrence as well as a paucity of targets for endocrine and anti-HER2 treatment. Some obstacles to surgery and anesthesia in cancer treatment, such as physiological disturbances, tumor-related symptoms and toxicity in traditional chemotherapy treatment, do exist. Consequently, therapy for TNBC poses challenges that emphasize the restricted effect of systemic chemotherapy. The appropriate combination of surgical and anesthetic procedures and medications can reduce perioperative inflammatory and immune changes that could contribute to improved results for cancer patients. (33)

The repurposing strategy of tramadol was applied to the development of therapy for breast cancer. The therapeutic blood levels of tramadol in adults range from 0.1 to 0.3 mg/L, the toxic level is between 1 and 2 mg/L, and the lethal concentration is higher than 2 mg/L. (34) Our working dosages were based on the values of IC_50_ (0.8 and 1.1 mg/mL for MDA-MB-231 and MCF-7, respectively) which were higher than the clinical therapeutic level of tramadol for a centrally acting analgesic but were consistent with the study of Kim and colleagues (8) for anti-tumorigenic effects on breast cancer cells. Furthermore, Kim and colleagues (35) investigated an *in-vivo* experiment for confirmation the anti-tumor effect of tramadol in xenograft mice with orthotopic inoculation of MCF-7 cells and revealed the clinical dosage of tramadol (1.5 and 3 mg/kg/day), could impede tumor growth, the tumor size and weight compared to the control or morphine groups. Kim et al. alternatively provided *in-vivo* evidences of achievable dose of tramadol in clinical settings.

Here, we demonstrated a novel therapeutic strategy by combining tramadol with doxorubicin for the effective treatment of breast cancer. Our results indicate a synergistic effect of tramadol and doxorubicin in breast cancer, despite the predominantly analgesic purpose of tramadol rather than its application in adjuvant chemotherapy. The therapeutic concentrations of 24 μM doxorubicin were decreased to 0.3 μM (at 0.26 mg/mL tramadol) in MDA-MB-231 cells and 5.1 μM doxorubicin were decreased to 1.2 μM (at 0.24 mg/mL tramadol) in MCF-7 cells, which translated to a diminished adverse-effect profile and lower risk of doxorubicin-induced resistance in metastatic breast cancer. However, further *in vivo* and clinical studies are necessary to determine the actual clinical dose of tramadol and doxorubicin in breast cancer, especially for TNBC. In addition, we anticipate that future studies of efficient systems pharmacology platforms containing absorption, distribution, metabolism, and excretion properties will elucidate the optimal dosage of tramadol for the combination therapy.

Turning to the different toxicity of tramadol in MCF-7 and MDA-MB-231 cells, the two cells are infiltrating duct/breast cancer cells, but each own many phenotype/genotype differences: MCF-7 is hormone-dependent (expression of both ER and PR), while MDA-MB-231 is triple negative. Furthermore, MCF-7 cells express markers of the luminal epithelial phenotype, while MDA-MB-231 cells show high expression of vimentin (**Figure 5**), a known marker of the mesenchymal phenotype. The results of xenograft mouse model through MCF-7 by Kim and colleagues (35) have shown that tramadol may have receptor-specific anti-tumor effects through ER, PR and TRPV1. In comparison, MDA-MB-231 cells lack hormone receptors, and the toxicity caused by tramadol must be different from that of MCF-7 cells.

Moreover, dynamic changes in cancer cell plasticity are derived from EMT, which enables tumor cell mobilization and distant metastases. (36) It is clear that the initiation of invasion and metastasis of TNBC, and the resultant cancer mortality, is attributable to EMT progression. (37) HIF-1α expression is highly induced in hypoxic environments in MDA-MB-231 cells. Thus, hypoxia-induced EMT and HIF-1α expression can regulate the expression of angiogenesis and promote tumor cell metastasis. (16, 38) We found that tramadol, in some way, interfered with the transformation of MDA-MB-231 and MCF-7 cells into the mesenchymal state, which has implications for providing regulatory EMT capacities, and eventually suppressed the migration, colony formation and invasion of breast cancer cells. Both hypoxia and tramadol induced HIF-1α expression; however, no further induction by tramadol in hypoxic MDB-MA-231 and MCF-7 cells was found. HO-1 proteins were induced by tramadol or hypoxia but suppressed by tramadol in hypoxic MDB-MA-231 and MCF-7 cells. The HO-1 gene is a target gene of HIF-1α and tramadol and hypoxia potentially modified HIF-1α expression. Further elucidation of the mechanisms of HIF-1α protein induction by tramadol is required.

On the other hand, one characteristic of cancer is the uncontrolled proliferation of tumor cells caused by the abnormal activity of various cell cycle proteins. Many studies have pointed out that cyclin D1 is overexpressed in more than 50% of breast cancers, and the amplification of the Cyclin D1 gene is related to poor prognosis of patients. (39, 40) In recent years, *in vitro* and *in vivo* studies have identified the new role of cyclin D1 as a controller of cellular invasiveness and aggressiveness. (41, 42) The progression of the G1 phase of the cell cycle is mainly controlled by cyclin D1. Cyclin D1 is located in the nucleus and reaches its highest level before the S phase. At the end of the G1 phase and after entering the S phase, cyclin D1 is exported to the cytoplasm and degraded by the ubiquitin-proteasome system. In our study, tramadol induced a significant dose-dependent decrease in the level of cyclin D1 protein in MCF-7 cells and had no significant effect on MDA-MB-231 cells. This result is consistent with our cell cycle profile. The highest dose of tramadol in MCF-7 cells was shown to induce the S phase and reduce the G1 phase. It also increased cell cycle arrest in the subG1 and G2/M phase. In MDA-MB-231 cells, it was found that the G1, subG1 phase were tramadol-induced without affecting the S phase, and unlike MCF-7, its G2/M phase was reduced. This result indicated that tramadol has different effects on the cell cycle of MCF-7 and MDA-MB-231.

In conclusion, tramadol inhibited cell growth, cell migration, colony formation and invasion; regulated the EMT process; and induced a cytotoxic effect in MDA-MB-231 and MCF-7 cells. These findings suggest that tramadol might be a candidate for combination therapy for breast cancer, especially for TNBC. In addition, co-administration of tramadol might reduce the effective dosage of doxorubicin, which indicates a promising treatment strategy in clinical practice for breast cancer patients.

## Data Availability Statement

The original contributions presented in the study are included in the article/supplementary material. Further inquiries can be directed to the corresponding author.

## Author Contributions

Y-HH and S-HS performed experiments, collected data, and wrote the manuscript. Z-SW performed experiments and prepared the figures. S-MH provided cell lines for the experiments and was assistant in data analysis. S-YL collected data. Z-FW designed the experiments, wrote the manuscript, and supervised the work. All authors contributed to the article and approved the submitted version.

## Funding

This work was supported by grants from the Ministry of Science and Technology (MOST109-2635-B016-001), Taiwan, Republic of China.

## Conflict of Interest

The authors declare that the research was conducted in the absence of any commercial or financial relationships that could be construed as a potential conflict of interest.

## Publisher’s Note

All claims expressed in this article are solely those of the authors and do not necessarily represent those of their affiliated organizations, or those of the publisher, the editors and the reviewers. Any product that may be evaluated in this article, or claim that may be made by its manufacturer, is not guaranteed or endorsed by the publisher.
